# Integrative profiling of metabolome and transcriptome of skeletal muscle after acute exercise intervention in mice

**DOI:** 10.3389/fphys.2023.1273342

**Published:** 2023-10-06

**Authors:** Xing Ye, Renyi Liu, Zhixian Qiao, Xiaocui Chai, Yan Wang

**Affiliations:** ^1^ School of Physical Education, China University of Geosciences (Wuhan), Wuhan, China; ^2^ Institute of Hydrobiology, Chinese Academy of Sciences, Wuhan, China

**Keywords:** acute exercise, metabolome, transcriptome, skeletal muscle, quadriceps, mice

## Abstract

This study aims to explore the molecular regulatory mechanisms of acute exercise in the skeletal muscle of mice. Male C57BL/6 mice were randomly assigned to the control group, and the exercise group, which were sacrificed immediately after an acute bout of exercise. The study was conducted to investigate the metabolic and transcriptional profiling in the quadriceps muscles of mice. The results demonstrated the identification of 34 differentially expressed metabolites (DEMs), with 28 upregulated and 6 downregulated, between the two groups. Metabolic pathway analysis revealed that these DEMs were primarily enriched in several, including the citrate cycle, propanoate metabolism, and lysine degradation pathways. In addition, the results showed a total of 245 differentially expressed genes (DEGs), with 155 genes upregulated and 90 genes downregulated. KEGG analysis indicated that these DEGs were mainly enriched in various pathways such as ubiquitin mediated proteolysis and FoxO signaling pathway. Furthermore, the analysis revealed significant enrichment of DEMs and DEGs in signaling pathways such as protein digestion and absorption, ferroptosis signaling pathway. In summary, the identified multiple metabolic pathways and signaling pathways were involved in the exercise-induced physiological regulation of skeletal muscle, such as the TCA cycle, oxidative phosphorylation, protein digestion and absorption, the FoxO signaling pathway, ubiquitin mediated proteolysis, ferroptosis signaling pathway, and the upregulation of KLF-15, FoxO1, MAFbx, and MuRF1 expression could play a critical role in enhancing skeletal muscle proteolysis.

## 1 Introduction

Skeletal muscle, being the most abundant tissue in the body, is indispensable for movement as the body relies on the contraction and relaxation of skeletal muscle. Furthermore, a key predictor of mortality is muscle mass quality (Baskin et al., 2015). The skeletal muscle plays a crucial role in maintaining the balance of metabolic processes, making it one of the primary sites for glucose disposal (DeFronzo and Tripathy, 2009). Unhealthy lifestyles and habits have led to an alarming increase in the incidence of various chronic diseases, imposing a significant economic burden on healthcare systems worldwide (Kim et al., 2016; Farr and Khosla, 2019; Castellanos et al., 2020). It is well known that exercise, as a stressor, has a significant impact on physical function (Cook et al., 2012; Wang et al., 2021). Moreover, exercise is a major factor in the extensive metabolic and molecular remodeling of skeletal muscles (Egan and Zierath, 2013). The skeletal muscle undergoes various adaptations in response to exercise, including the synchronization of muscle contraction and ATP production, as well as the utilization of energy triggered by mechano- and other metabolic sensors (Egan and Zierath, 2013; Camera et al., 2016; Fan and Evans, 2017).

Studies have shown that regular physical activity enhances exercise performance, induces increased skeletal muscle protein synthesis, inhibits skeletal muscle protein degradation (Qiu et al., 2014), improves mitochondrial function, and subsequently promotes metabolic homeostasis in the body (Shen et al., 2021). However, intensive or prolonged exercise can trigger an oxidative imbalance, which can cause oxidative damage and lead to muscle fatigue (Reid et al., 1992; O'Neill et al., 1996). If the ability to remodel skeletal muscles decreases and their quality deteriorates, it can affect the bodyʼs motor function and increase the risk of falls and injuries. The enhancement of muscle function is achieved through improved the exercise capacity and the optimized metabolic functions. Despite significant efforts to uncover the comprehensive changes in muscle caused by exercise, there are still many regulators of skeletal muscle that have yet to be discovered.

Transcriptomic profiling is a powerful technique that allows for the identification of intricate gene expression patterns (Wang et al., 2009). On the other hand, metabolomics serves as a valuable tool in detecting and elucidating changes in metabolites within a biological system under varying conditions (Dettmer et al., 2007), but few studies have investigated the comprehensive transcriptomic and metabolomic profile of exercise muscular physiology. The objective of this study was to evaluate and analyze the transcriptional and metabolic networks of skeletal muscle that are regulated by exercise in mice. By doing so, we were able to gain insights into the mechanisms through which exercise intervention impacts the overall metabolic balance of skeletal muscle.

## 2 Material and methods

### 2.1 Animals

The study used C57BL/6 male mice were 9 weeks old and weighing 22.1 ± 1.3 g. The mice utilized in this study were housed in the animal facilities located at Hubei Provincial Center for Disease Prevention and Control in China, where they received a standard photoperiod of 12-h light/12-h dark cycles. Mice were housed under standard conditions, with access to food and water unrestricted, at a temperature of 21°C ± 1°C. After 1 week of the acclimation period, the animals were randomly assigned to the control or exercise groups. All animal experiments were approved by the local Animal Care and Use Committee and performed following the recognized guidelines for laboratory animal care and management. Ethical approval was granted by the Academic Integrity and Scientific Research Ethics Committee of China University of Geosciences (Wuhan).

### 2.2 Aerobic capacity test and treadmill exercise protocol

This study utilized a mouse treadmill, following the method previously mentioned by Kruger et al. (2009) and Kemi et al. (2002). To minimize the stress caused by the equipment, the animals were provided with 1 week of exercise adaptation prior to undergoing the exercise test. Each animal underwent an incremental exercise test to exhaustion at least 4 days prior to commencing the experiments to measure their maximal oxygen uptake ( $\dot{V}$ O_2_max) and maximal running speed (Vmax). The mice were acclimated in the treadmill chamber for 10 min before undergoing a continuous, progressive exercise test until exhaustion. The test involved a 5-min warm-up at a speed of 0.20 m/s, followed by an increase in running speed by 0.05 m/s every 3 min until the mice reached a state of exhaustion. The animals were randomly divided into two groups, each consisting of six animals. The mice from the control group were exposed to the noise produced by the treadmill without engaging in actual running. On the other hand, the animals in the exercise group underwent a single running test until exhaustion at an intensity of 80% VO_2_max, which corresponded to a speed of 0.40 ± 0.30 m/s.

### 2.3 Tissue samples

After conducting the exercise tests, the animals were administered anesthesia, and then euthanized using cervical dislocation. The muscle samples used in this study referenced the experimental conditions described previously (Song et al., 2023). The posterior thigh of mice was sterilized, and a skin incision was made. The m. gluteus superficialis and the tensor fasciae latae muscle were identified. A sample was taken from the exposed quadriceps femoris muscle. The quadriceps were weighed; mass was normalized to body mass. To maintain its integrity, the specimen was promptly cooled in a pre-chilled diethylpyrocarbonate (DEPC) solution at a temperature of 4°C, effectively eliminating any superficial blood and hair. Then, the muscular tissue was desiccated using filter paper, carefully placed into a cryopreservation tube, and promptly placed in liquid nitrogen for subsequent examination.

### 2.4 Experimental methods of metabolomics

Retrieve the sample from liquid nitrogen and place it on ice to thaw until it reaches a state where it can be easily cut (all subsequent steps were performed on ice). Chop and mix the samples, and weigh the samples at 20 mg (±1 mg) into the corresponding numbered centrifuge tubes. Add a steel ball using forceps, homogenize for 20 s with a ball mill (30 HZ), and centrifuge the sample at 3,000 r/min for 30 s at 4°C (the time can be increased according to the actual homogenization). After centrifugation, add 400 μL of 70% methanolic water internal standard extract, centrifuged at 2,500 r/min for 5 min, and let stand on ice for 15 min. Centrifuge at 12,000 r/min for 10 min at 4°C, transfer 300 μL of the supernatant to another centrifuge tube with a corresponding number, and let it stand in a −20°C refrigerator for 30 min; Under 4°C conditions, centrifuge again at 12,000 r/min for 3 min, transfer 200 μL of the supernatant into a lined tube inside the corresponding sample bottle for analysis on the machine. The samples were then analysed using ultra-performance liquid chromatography-tandem mass spectrometry (UPLC-MS/MS). Mass spectroscopy data processing was performed using Analyst software (version 1.6.3). Orthogonal projections to latent structures-discriminant analysis (OPLS-DA) were employed to analyze the differentially expressed metabolites (DEMs). DEMs were screened based on a combination of fold change, *t*-test *p*-value, and variable importance in the projection (VIP) values from the OPLS-DA model. The screening criteria were set as VIP >1 and *p*-value <0.05. Use R language to perform OPLS-DA to determine the magnitude of differences between samples. Subsequently, metabolic pathway enrichment analysis of DEMs was performed.

### 2.5 RNA-seq experimental method

Total RNA was isolated from skeletal muscle using the RNeasy Mini Kit (Qiagen, Hilden, Germany), and the quantity and purity were determined with a UV-Vis spectrophotometer (ND-1000, Nano-Drop Technologies, Rockland, United States). During RNA separation, the RNase-free DNase set (Qiagen, Hilden, Germany) was used to perform on-column DNase digestion, ensuring the elimination of any potential DNA contamination. The transcriptomic sequencing (RNA-seq) was performed using Illumina Novaseq 6000 (Illumina, CA) to construct and sequence libraries. The high-quality data were aligned to a transcript reference obtained from the mouse reference genome (version mm10), through Bowtie2 default parameters (Langmead and Salzberg, 2012). Transcript abundance was normalized and quantified utilizing RSEM (RNA-Seq by Expectation Maximization) (Li and Dewey, 2011). Differentially expressed genes (DEGs) were detected using a threshold of Fold Change ≥2 and FDR <0.01. Subsequently, the obtained DEGs were subjected to Gene Ontology (GO) and Kyoto Encyclopedia of Genes and Genomes (KEGG) functional annotation and enrichment analysis.

### 2.6 Validation of DEGs by quantitative real-time PCR

To validate the reliability of RNA-seq data, a subset of 5 differentially expressed genes (DEGs) was selected for quantitative real-time PCR (qPCR) using the primers provided in Table 1. The High-capacity cDNA reverse transcription kit (Applied Biosystems) was used according to the manufacturerʼs instructions to synthesize cDNA from the RNA samples. The cDNA was obtained using the T100 PCR thermal cycler (Bio-Rad Laboratories, Munich, Germany) and used for qPCR. The iQ SYBR Green Supermix (Bio-Rad Laboratories, Munich, Germany) was utilized for qPCR to measure the mRNA expression. The amplification products were quantified using an iCycler (Bio-Rad Laboratories, Munich, Germany). The reaction conditions included an initial denaturation at 95°C for 3 min, followed by 42 cycles of denaturation at 95°C for 15 s, annealing at 61°C for 30 s, and elongation at 72°C for 30 s. The mRNA expression levels were normalized to the expression of the housekeeping gene *β*-actin. The results were analyzed using the 2^−Δ(ΔCt)^ method and presented as fold changes relative to the control group.

**TABLE 1:** Primer information for RT-qPCR.

Gene	Primer sequence (5′ to 3′)	
	F	R
MAFbx	CAG​CTT​CGT​GAG​CGA​CCT​C	GGC​AGT​CGA​GAA​GTC​CAG​TC
KLF-15	GAG​ACC​TTC​TCG​TCA​CCG​AAA	GCT​GGA​GAC​ATC​GCT​GTC​AT
UCP3	CTG​CAC​CGC​CAG​ATG​AGT​TT	ATC​ATG​GCT​TGA​AAT​CGG​ACC
Atf3	GAG​GAT​TTT​GCT​AAC​CTG​ACA​CC	TTG​ACG​GTA​ACT​GAC​TCC​AGC
Sap30	CAA​CTC​TGT​TGT​TTG​CGG​GAG	AGA​TGC​CTT​GCA​CTC​TTA​TCC​A
β-actin	GTG​ACG​TTG​ACA​TCC​GTA​AAG​A	GCC​GGA​CTC​ATC​GTA​CTC​C

### 2.7 Combined analysis of transcriptome and metabolome

Conjoint Analysis of DEGs and DEMs is conducted based on the relative content data from transcriptomics and metabolomics. Enrichment analysis is performed on the significantly enriched KEGG pathways for both DEGs and DEMs. This analysis identifies the pathways that are jointly enriched by DEGs and DEMs, and these pathways are plotted as bar graphs.

### 2.8 Statistical analysis

This study presents the mean values and standard errors of the mean, which were determined. The data was tested for normality using the Shapiro-Wilk test, and the results indicated that the data followed a normal distribution. A one-way ANOVA test was conducted to analyze the differences between the groups, and the results were considered statistically significant at *p* < 0.05. All statistical analyses were performed using SPSS 26.0 software.

## 3 Results

### 3.1 Screening for differential metabolites

**FIGURE 1:**
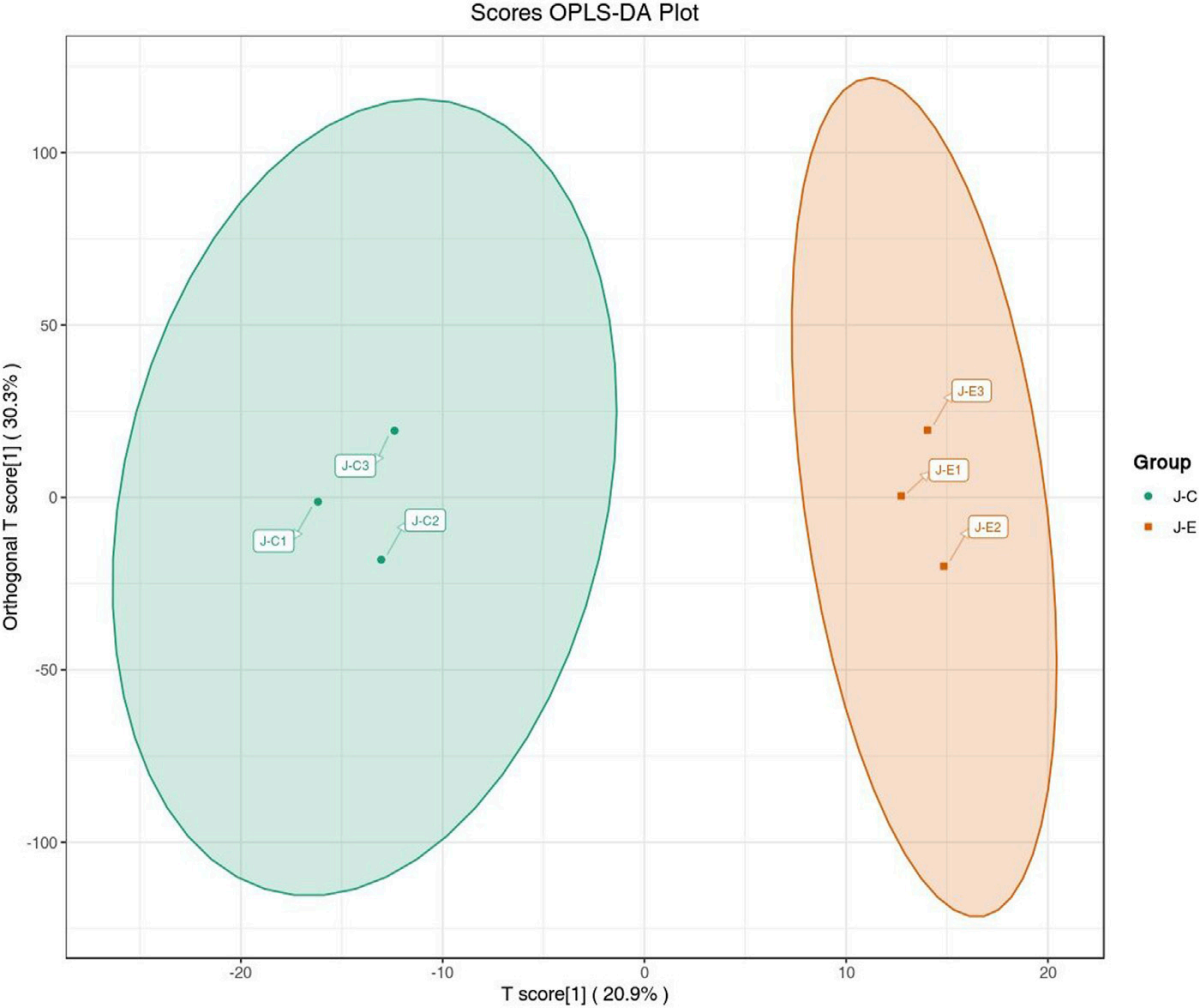
Score plot of the OPLS-DA. The abscissa indicates the predicted principal component; the ordinate indicates the orthogonal principal component; the percentage indicates the explanation rate of this component to the data set. Each point in the graph indicates a sample, and samples of the same group are represented using the same color.

**FIGURE 2:**
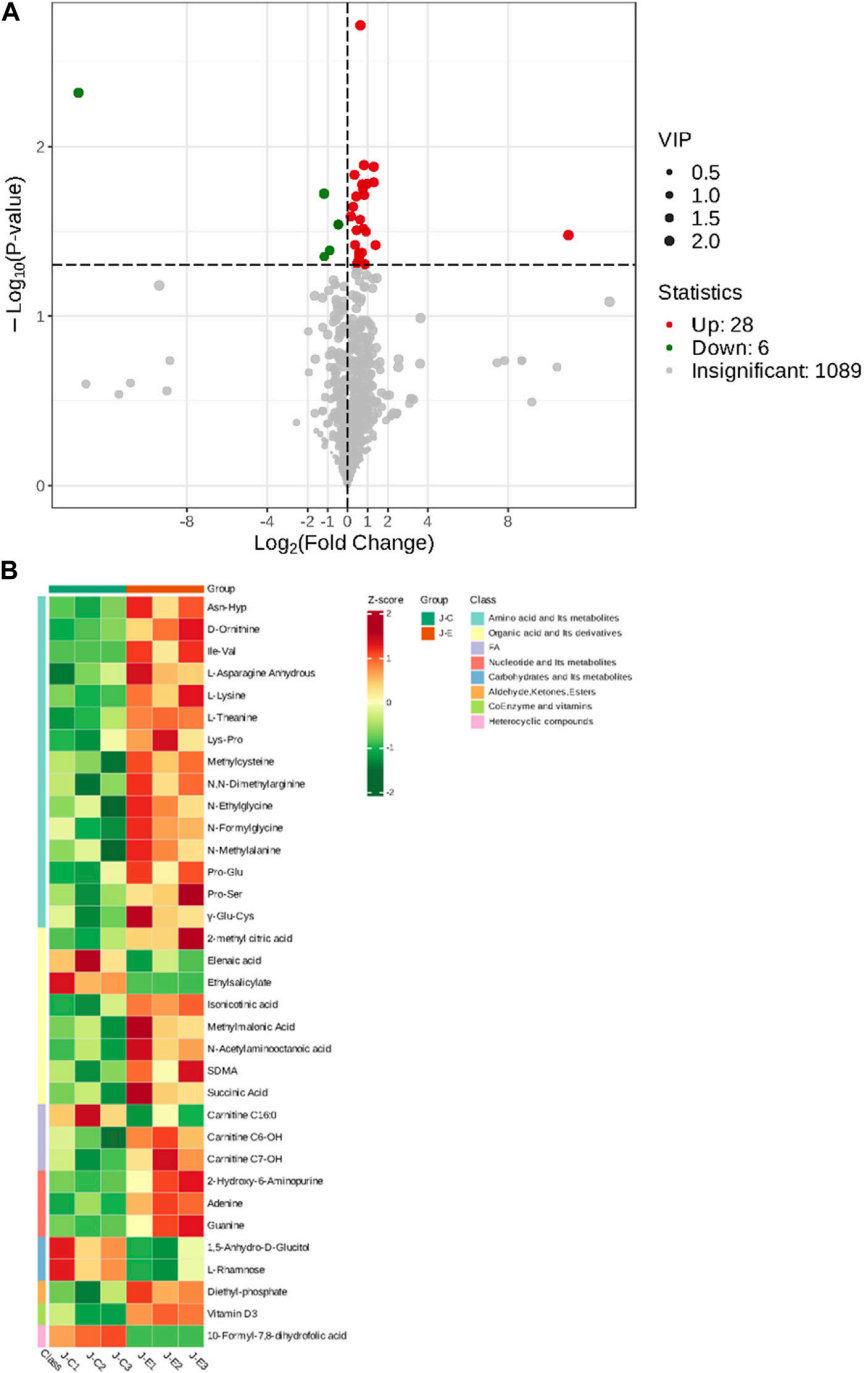
Screening of Differentially Expressed Metabolites. **(A)**: Volcano plot of differentially expressed metabolites. Each point in the volcano plot represents a metabolite, with green dots representing downregulated differential metabolites, red dots representing upregulated differential metabolites, and gray representing metabolites that are detected but not significantly different. The abscissa represents the logarithmic value (log2FC) of the relative abundance difference of a metabolite between the two groups of samples. The larger the absolute value of the abscissa, the greater the relative abundance difference of the metabolite between the two groups of samples. Under the filtering conditions of VIP + FC + *p*-value, the ordinate represents the significance level of the difference (-log10*p*-value), and the size of the dots represents the VIP value. **(B)**: Clustering heat map of the metabolites. The abscissa is used to display the names of samples, and the ordinate on the right is used to display the names of metabolites. Different colors are filled with different values obtained after normalization for different relative contents. Red indicates high content; green indicates low content.

The OPLS-DA model showed that the two groups of samples could be separated, indicating that the intervention of an acute bout of exercise caused changes in skeletal muscle metabolism in rats (Figure 1). The T score was 20.9% and the orthogonal T score was 30.3% indicating significant differences in metabolites between the experimental group and the control group. The fold change was combined with the VIP values of the OPLS-DA model to filter the DEMs. In the exercise group and the control group, a total of 34 DEMs were identified, comprising 28 upregulated metabolites and 6 downregulated metabolites (Figure 2). These include 15 amino acids and its metabolites, 8 organic acids and its derivatives, 3 nucleotide and its metabolites, 3 fatty acyls, 2 carbohydrates and its metabolites, 1 heterocyclic compound, 1 coenzyme and vitamin, and 1 aldehyde, ketone, and ester.

### 3.2 Signaling pathway analysis of differential metabolites

**FIGURE 3:**
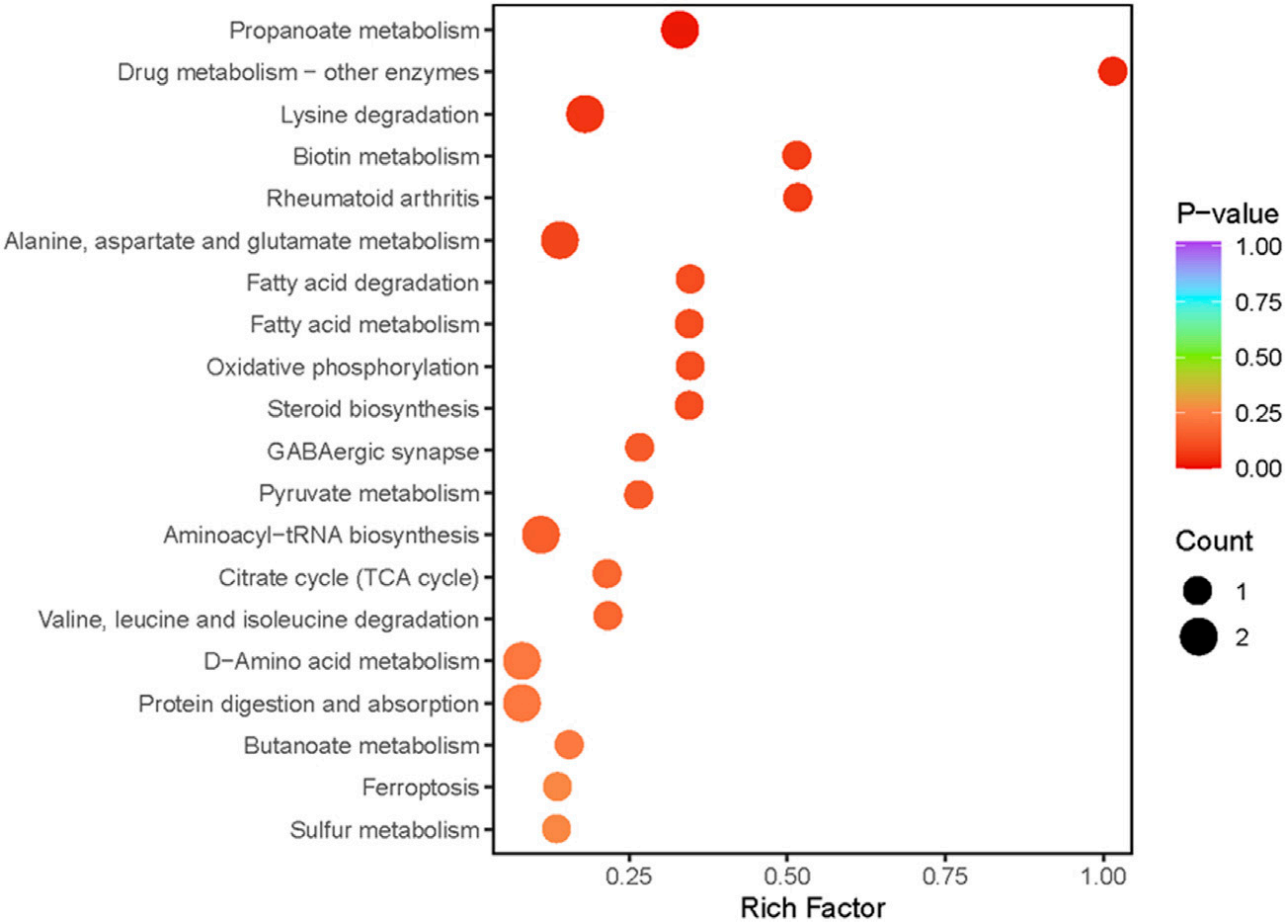
Pathway analysis of differentially expressed metabolites. The abscissa indicates the Rich Factor for each pathway, the ordinate is the pathway name (sorted by *p*-value), and the color of the dot reflects the *p*-value size, the redder the enrichment is, the more significant the enrichment is. Dot size relates to the number of differentially expressed metabolites.

Metabolic pathway analysis was conducted on the DEMs between the exercise group and the control group, and a significant enrichment pathway for metabolites was obtained using a hypergeometric distribution algorithm (*p* < 0.05) (Figure 3). The top 20 pathways in terms of *p*-value were selected for display from smallest to largest and made into a bubble chart. The DEMs were mainly enriched in propanoate metabolism, protein digestion and absorption, d-amino acid metabolism, lysine degradation, citrate cycle, alanine, aspartate and glutamate metabolism, ferroptosis, pyruvate metabolism and aminoacyl-tRNA biosynthesis.

### 3.3 Screening of differential gene expression

**FIGURE 4:**
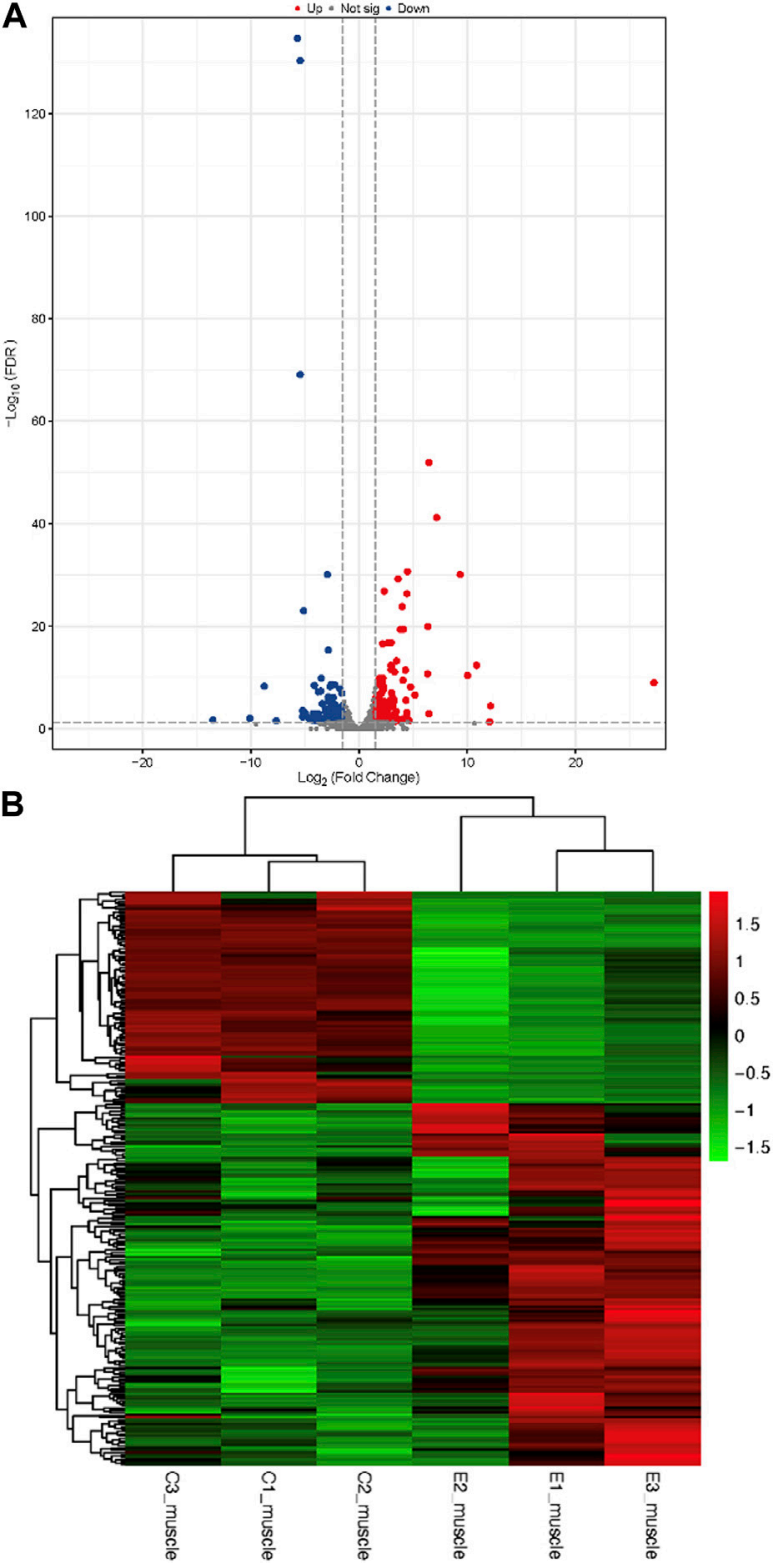
Identification of differentially expressed genes **(A)**: Volcano plots of differentially expressed genes. In a volcano plot, each dot represents a gene. Blue dots represent the downregulated genes, red dots represent the upregulated genes, and the gray dots represent the non-significant genes **(B)**: Heatmap of differentially expressed genes.

The raw data were obtained from 6 sequenced samples of skeletal muscle total RNA samples after sequencing by the transcriptome Illumina Novaseq 6000 sequencing platform. Using Fold Change≥2 and FDR<0.01 as criteria, we identified 245 DEGs in the exercise group compared with the control group. Among them, 155 upregulated genes and 90 downregulated genes accounted for 63% and 37% of the total DEGs, respectively (Figure 4). Furthermore, we have identified 10 key genes that are highly associated with skeletal muscle (Table 2).

**TABLE 2:** Results of analysis of differential expression of key candidate genes.

Gene	Description	FDR	Up/down
MuRF1	Muscle RING-finger protein-1	1.82287E-09	up
MAFbx	Muscle Atrophy F-box protein	5.30082E-05	up
KLF-15	Kruppel-like factor 15	0.00078	up
FoxO1	Forkhead box O1	1.69647E-05	up
Tigar	Trp53 induced glycolysis regulatory phosphatase	2.71345E-09	down
Srl	Sarcalumenin	1.9239E-135	down
Sirt6	Sirtuin 6	0.00903	up
UCP3	Uncoupling protein 3 (mitochondrial, proton carrier)	1.09267E-08	up
Atf3	Activating transcription factor 3	1.10252E-52	up
HSP70	Heat shock protein70	6.8673E-42	up

### 3.4 GO and KEGG analysis of differentially expressed genes

**FIGURE 5:**
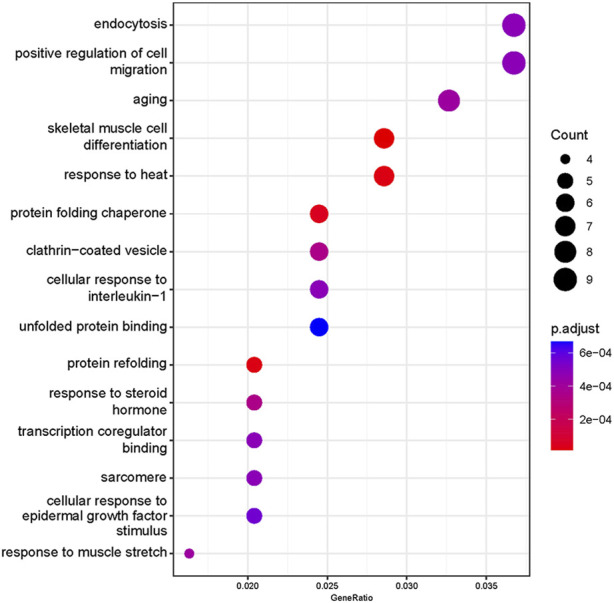
Gene Ontology enrichment analysis of differentially expressed genes. The abscissa is the ratio of the number of genes to all genes for the term, ordinate shows the GO terms, the size of the bubble represents the number of genes enriched in the GO terms, and color showed the q-value of GO terms.

**FIGURE 6:**
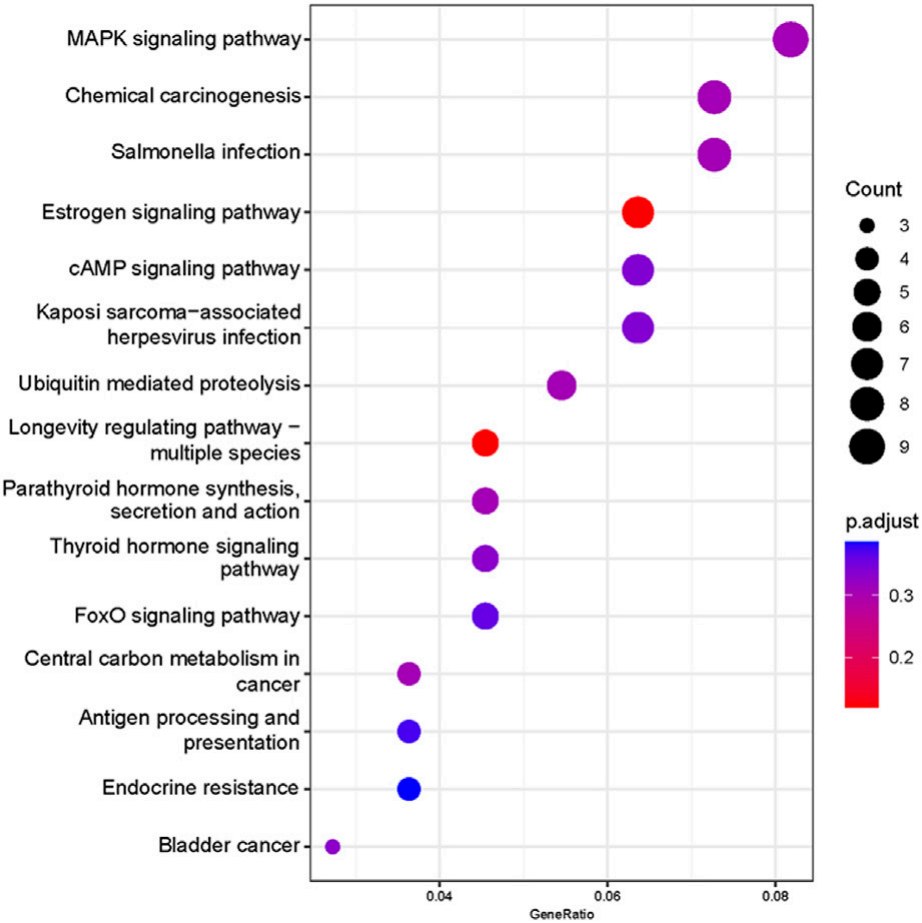
Functional annotation of the KEGG pathway for differentially expressed genes. Ordinate: nomenclature of the KEGG signaling pathway; Abscissa: gene numbers that annotated this pathway and the ratio of KEGG in all genes that annotated relative to the total KEGG.

Based on our screened DEGs, the top 15 GO Terms in *q*-value ranking from GO functional analysis were selected for presentation and bubble plotted (Figure 5). The DEGs were annotated to skeletal muscle cell differentiation, protein folding chaperone, aging, response to muscle stretch, positive regulation of cell migration and response to heat in the biological process functional classification. The 245 differential genes were subjected to KEGG enrichment analysis, and the results showed that a total of 238 signaling pathways were enriched. The top 15 enriched KEGG signaling pathways were selected for display (Figure 6). These include the ubiquitin mediated proteolysis, longevity regulating pathway, cAMP signaling pathway, FoxO signaling pathway, and MAPK signaling pathway.

### 3.5 Integrative KEGG pathway analysis of the transcriptome and metabolome

**FIGURE 7:**
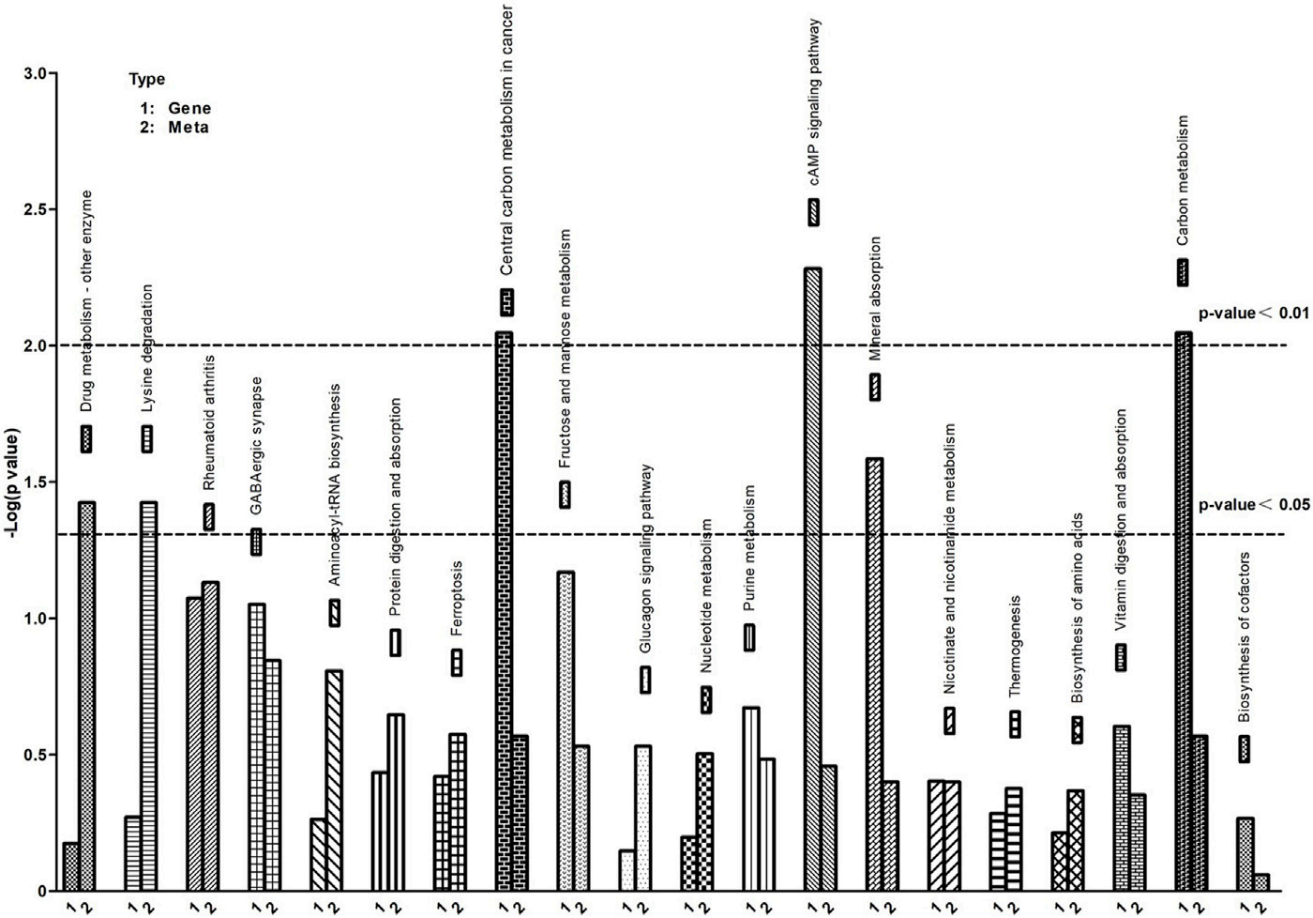
KEGG pathway enrichment of differential genes and metabolites. The abscissa represents signaling pathways, the ordinate represents enriched *p*-values, expressed as -log (*p*-value).

To further understand the correlation between metabolites and genes involved in the same biological process, we conducted a comprehensive analysis of transcriptomic and metabolomic data, resulting in the identification of 20 KEGG signaling pathways (Figure 7). The KEGG pathways predominantly enriched in differentially expressed metabolites and genes include protein digestion and absorption, biosynthesis of amino acids, cAMP signaling pathway, protein digestion and absorption and ferroptosis, among others.

### 3.6 Quantitative real-time PCR

**FIGURE 8:**
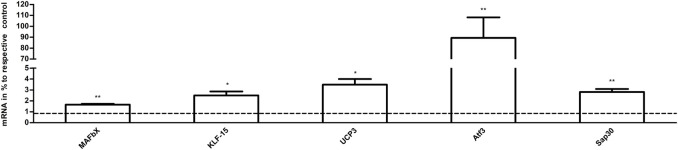
The effects of acute exercise on the gene expression of muscle. Compared with the control group, the mRNA expression in the exercise group. The relative levels of target gene mRNA expression were normalized against the mRNA expression of internal housekeeping gene (β-actin). The expression of *β*-actin mRNA was not significantly different between the tested groups allowing a direct comparison (data not shown). Note that columns and error bars represent (mRNA in % to respective control ±SEM), and levels of significance (^*^ *p* < 0.05, ^**^ *p* < 0.01, n = 3) are indicated.

The results of qPCR analysis in the samples are graphically represented in Figure 8. The levels of mRNA in the exercise mice were analyzed and compared to those of control animals. The transcript levels of MAFbx, Atf3, and Sap30 (*p* < 0.01 vs. CG, n = 3), KLF-15 and UCP3 (*p* < 0.05 vs. CG, n = 3) were significantly increased in the exercise group. The results of RNA-Seq are consistent with these findings.

## 4 Discussion

Skeletal muscle, a crucial component in controlling movement, plays a fundamental role as the primary regulator of metabolism in various body systems. Its susceptibility to the metabolic regulations imposed by exercise is particularly notable. To the best of our knowledge, this study represents the first investigation that employs transcriptomics and metabolomics to extensively explore the collective effects of acute exercise on skeletal muscle. This study identified a total of 34 metabolites that exhibited differential regulation. These metabolites are primarily associated with several metabolic pathways, including the citrate cycle (TCA cycle), oxidative phosphorylation, and Valine, leucine and isoleucine degradation. This transcriptomics analysis unveiled 245 differentially expressed genes. The KEGG pathways involved in this study include FoxO, ubiquitin mediated proteolysis signaling pathway. By conducting a joint analysis of transcriptomics and metabolomics, 20 enriched KEGG pathways were identified, including biosynthesis of amino acids, ferroptosis, protein digestion and absorption signaling pathway. Based on the aforementioned findings, we focus on a comprehensive analysis to investigate the potential mechanism by which exercise regulates skeletal muscle function.

To begin with, the results of metabolomics in this study demonstrated a significant increase in succinate levels within the TCA cycle following acute exercise, as illustrated in Figure 3. Succinate, being an integral component of the TCA cycle, can accumulate when the flow of succinic acid within the cycle is hindered. The metabolic dysfunction in the TCA cycle can lead to a deterioration in physiological function. Succinate is generated within the mitochondria and its main destiny upon transition to an aerobic environment is to undergo oxidation at the same location. Consequently, in a scenario where aerobic metabolism significantly outweighs anaerobic metabolism, succinate will not accumulate. However, the accumulation of succinate under aerobic conditions can occur if oxygen cost far exceeds the individual’s maximal oxygen uptake (VO_2_max) and anaerobic energy production is significant (Hochachka and Dressendorfer, 1976). In the analysis of metabolic pathways, succinate is not only closely related to the TCA cycle, but is also involved in various other metabolic pathways, including oxidative phosphorylation (Zhao et al., 2017).

The TCA cycle is an important pathway for intracellular oxidative phosphorylation to produce ATP, and mitochondria are the main site where the TCA cycle takes place. Moderate-intensity exercise can improve mitochondrial function, whereas both high-intensity and exhaustive exercise may disrupt mitochondrial structure and cause mitochondrial disorders (Tondera et al., 2009; Schoepe et al., 2012). In particular, skeletal muscle is an important site for the uptake and utilization of fatty acids, and mitochondria play an important role in cellular energy metabolism, being the main site for energy conversion, the TCA cycle, and oxidative phosphorylation. The mitochondria undergo pathological changes following acute exercise, leading to impaired function (Magalhaes et al., 2013). As a result, the cellular capacity for oxidative phosphorylation is affected. This implies that the disruption of mitochondria by acute exercise may lead to an inadequate energy supply and adversely affect the organism.

Disruptions in mitochondrial function could be related to amino acid pool imbalances and altered amino acid metabolism (Naseer et al., 2020). The fascinating finding in this study was that strenuous exercise resulted in the degradation of amino acids such as valine, leucine, isoleucine, and so on (as depicted in Figure 3), while amino acids such as glycine and serine increased, demonstrating that the body undergoes degradation and production of proteins associated with the injury zone immediately after the onset of the injury. It is believed that succinate is produced as a result of the catabolism of amino acids. The findings of this study demonstrated that the levels of 15 amino acids and their derivatives, such as l-asparagine anhydrous, l-lysine, d-ornithine, pyroglutamic acid, and n, n-dimethylarginine and so on, were upregulated following an acute bout of intensive exercise. These changes in metabolites involve pathways such as d-amino acid metabolism, pyruvate metabolism, propanoate metabolism, and fatty acid degradation and so on. Besides, a source of anaerobic energy may be generated by amino acid catabolism (Hochachka and Dressendorfer, 1976). The noteworthy elevation of amino acids *in vivo* following acute exercise suggests that the rate of acid production in the skeletal muscle of exercising mice exceeds the rate of elimination, there is an increase in muscle protein degradation, which results in the release of amino acids from muscle tissue into the bloodstream. This phenomenon could be a compensatory mechanism to provide amino acids as a substrate for energy production and gluconeogenesis during exercise. Exercise could stimulate the activity of key enzymes involved in amino acid catabolism (Harris et al., 2005).

The general consensus is that protein synthesis in skeletal muscle is suppressed during exercise, as evidenced by findings in both humans and other species (Rennie et al., 1980; Dohm et al., 1985). The results of transcriptomics in this study show that acute exercise could induce an increase in KLF-15, FoxO1, MAFbx, and MuRF1 expression (Table 2). KLF-15, a key transcription factor, not only inhibits skeletal muscle protein synthesis but also plays a crucial role in enhancing skeletal muscle proteolysis by promoting MuRF1 and MAFbx transcription, and stimulates FoxO1 (Kuo et al., 2013). FoxO1, in particular, is the main regulatory factor among all FoxO subtypes, expressed significantly in skeletal muscle. This study demonstrates that the activation of the FoxO signaling pathway is significantly enhanced, as evidenced by the data presented in Figure 6. In the process of KLF-15 mediated skeletal muscle degradation, FoxO plays a pivotal role as a transcription factor and serves as a central component of multiple degradation pathways. Participating in moderate or vigorous physical activity leads to an intensified generation of reactive oxygen species (ROS) (Vezzoli et al., 2016). The FoxO signaling pathway may be activated as a result of increased generation of ROS (Dodd et al., 2010). The importance of this heightened FoxO signaling lies in its role in increasing FoxO nuclear translocation, therefore leading to an intensified transcription of FoxO target genes, such as MAFbx and MuRF-1, which are directly involved in muscle catabolism (Bodine et al., 2001; Gomes et al., 2001; Crow, 2002), and MAFbx and MuRF-1 play a crucial role in specifically targeting proteins for degradation by the proteasome (Bodine et al., 2001; Gomes et al., 2001; Puthanveetil et al., 2013). In addition, we found that the HSP70, UCP3 and SIRT6 genes were significantly upregulated in skeletal muscle after exercise in this study. The increase in expression of the differential gene HSP70 can induce a protective anti-apoptotic response, alleviate membrane protein denaturation caused by injury stimuli, and protect the protein synthesis pathway in cells from damage (Mosser et al., 2000; Mokranjac et al., 2006). This high expression of UCP3 can reduce the release of ROS and prevent damage to the body (Nabben et al., 2008; Toime and Brand, 2010), while SIRT6 overexpression inhibits the protein degradation pathway related to muscle atrophy (Samant et al., 2017). However, their protective effects may not be sufficient to counteract the physical damage caused by acute exercise.

The ubiquitin-proteasome system (UPS) is the primary pathway for intracellular protein degradation (Meng et al., 2014). The importance of the ubiquitination level of proteins for quality control is further highlighted by the fact that approximately 80% of proteins in the body are degraded by the ubiquitin-proteasome system (Cromm and Crews, 2017). There is evidence that abnormal protein metabolism can lead to excessive activation of the ubiquitin-proteasome system, resulting in protein degradation (Cunha et al., 2012). MAFbx and MuRF-1, which are both downstream signaling proteins of FoxO, are widely recognized as crucial skeletal muscle-specific F-box-type ubiquitin ligases involved in protein ubiquitination (Bodine and Baehr, 2014). This study demonstrates an enhanced process of ubiquitin mediated proteolysis, as depicted in Figure 6. Previous studies have examined the expression of MAFbx and MuRF1 genes in skeletal muscle during exercise. Mascher et al. (2008) found that after one session of knee joint resistance exercise in sedentary young men, the mRNA content of MuRF1 increased by 4.8 times. Louis et al. (2007) had subjects perform running and resistance exercises and found increased expression of MuRF1 and MAFbx through a muscle biopsy.

UPS could regulate the degradation of proteins involved in iron metabolism, thereby modulating ferroptosis (Zhang et al., 2010; Horie et al., 2017). In this study, Figure 7 vividly depicts the crucial discovery that ferroptosis serves as a pivotal biological process, identified through KEGG pathway enrichment analysis of differential genes and metabolites. Ferroptosis, a novel type of cell death has been reported (Dixon et al., 2012). It is a specific mechanism characterized by lipid peroxidation due to iron overload, which leads to cell death (Conrad et al., 2016). Studies have shown that ferroptosis is associated with various cellular processes, such as iron homeostasis, redox homeostasis, and lipid metabolism (Yang et al., 2014; Ingold et al., 2018). UPS, as a key regulatory system for homeostasis of eukaryotic cells (Glickman and Ciechanover, 2002). Ferroptosis impacted muscle fiber differentiation and promoted muscle protein degradation. Interestingly, some ubiquitin ligases are also involved in ferroptosis occurrence (Meng et al., 2022), while myasthenia-specific MAFbx and MuRF1 expression increases with iron accumulation in the skeletal muscle of aging individuals (Bodine and Baehr, 2014; Huang et al., 2021).

Thus, take all together, this study identified multiple metabolic pathways and signaling pathways that were involved in the regulation of skeletal muscle, such as the TCA cycle, oxidative phosphorylation, protein digestion and absorption, the FoxO signaling pathway, ubiquitin mediated proteolysis, and ferroptosis signaling pathway. Additionally, the study demonstrated that acute exercise led to the upregulation of KLF-15, FoxO1, MAFbx, and MuRF1 expression, which could play a critical role in enhancing skeletal muscle proteolysis. However, the study mainly focused on the metabolic and transcription profiling of mouse muscle, and did not involve other potential biomarkers or mechanisms, such as protein spectrometry or post-transcriptional modifications of gene expression.

## Data Availability

The datasets presented in this study can be found in online repositories. The names of the repository/repositories and accession number(s) can be found below: https://www.ncbi.nlm.nih.gov/search/all/?term=PRJNA1002978.
